# Electrogenic Cardioversion

**DOI:** 10.14740/cr334w

**Published:** 2014-05-15

**Authors:** Joseph Theodore, Rajiv Ananthakrishna, Prabhavathi Bhat, Dattatreya PV Rao, Manjunath Cholenhally Nanjappa

**Affiliations:** a Sri Jayadeva Institute of Cardiovascular Sciences and Research, Jayanagar 9th Block, Bannerghatta Road, Bangalore, 560069, India

**Keywords:** Hyperkalemia, Atrial fibrillation, Cardioversion

## Abstract

Atrial fibrillation (AF) is the most common arrhythmia in clinical practice. Cardioversion for AF may be performed by either using direct current (DC) shock (electrical cardioversion) or using drugs (chemical cardioversion). Here we report a case of a patient with heart failure and AF, who reverted to the normal sinus rhythm on correction of hyperkalemia (electrogenic cardioversion). The patient maintained sinus rhythm during follow-up. We highlight the importance of serum potassium in patients with AF.

## Introduction

Hyperkalemia occurs in 1-10% [1-4] of patients hospitalized for diverse reasons. This condition occurs more commonly in patients with heart failure secondary to the use of angiotensin converting enzyme (ACE) inhibitors, aldosterone antagonists and in patients with impaired renal function. The effects of hyperkalemia on the action potential of cardiac muscle and thereby surface electrocardiograph (ECG) are well known. There have been a few case reports of atrial fibrillation (AF) getting transiently converted to sinus rhythm during treatment of hyperkalemia [5]. However the case reported herewith is unique in terms of maintenance of the normal sinus rhythm for a prolonged period after normalization of serum potassium levels.

## Case Report

**Figure 1:**
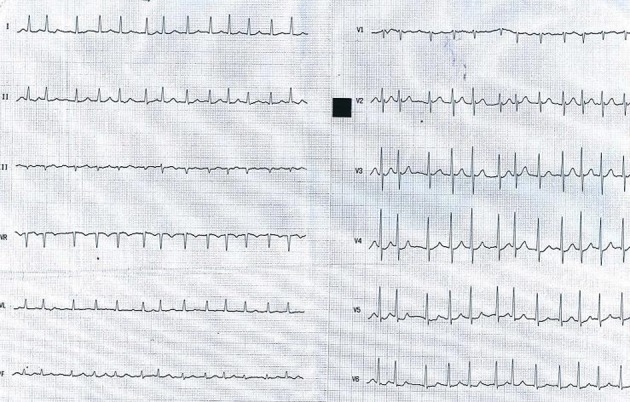
AF with fast ventricular rate at admission.

A 65-year-old woman with type 2 diabetes mellitus was admitted with left ventricular failure and accelerated hypertension (blood pressure of 180/100). She was a known case of ischemic dilated cardiomyopathy (DCM) with severe left ventricular (LV) dysfunction and long standing AF with fast ventricular rate (Fig. 1). Chest film showed pulmonary venous hypertension; two-dimensional transthoracic echocardiography showed dilated chambers with global systolic dysfunction with ejection fraction (EF) of 30%. Treatment was initiated with intravenous nitroglycerine infusion and frusemide. Subsequently, her blood pressure and failure symptoms were brought under control. During hospital stay, she developed profuse diarrhoea followed by severe hyperkalemia (serum potassium 8.3 mEq/L). Her complete blood count, renal and liver parameters were normal. Arterial blood gas analysis showed compensated metabolic acidosis.

**Figure 2:**
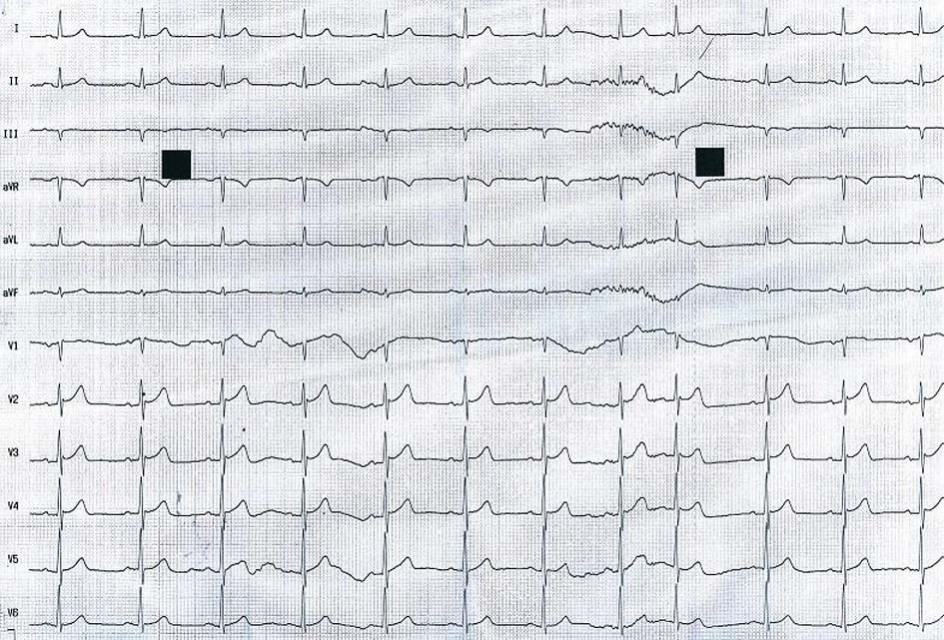
Sinus rhythm obtained at correction of hyperkalemia.

She was promptly treated with intravenous calcium gluconate, insulin dextrose infusion and salbutamol nebulisation. Serum potassium levels normalized to 5.4 mEq/L. ECG was repeated the next day, which showed sinus rhythm at 64 beats per minute. The patient was discharged on control of her failure symptoms after 5 days of hospital stay with aspirin, ACE inhibitors, low dose loop diuretics, beta blockers, digoxin and eplerenone. She had a CHADS-VASc score of 3 and was started on lifelong oral anticoagulation with warfarin to maintain INR of 2-3. ECG done at discharge and at 1 month follow-up revealed that the patient maintained normal sinus rhythm (Fig. 2).

## Discussion

The ECG changes of hyperkalemia have been well established. Severe hyperkalemia slows down the heart rate by suppressing the sino-atrial (SA) and atrio-ventricular (AV) nodal conduction rates. It also makes the atrial and ventricular myocardial cells refractory to impulse conduction. These changes are reflected in the surface ECG as tall T waves, prolonged PR interval, broad QRS complexes with AV and bundle branch blocks and finally as sine wave pattern [6].

Interestingly, previous reports have documented unusual cardiac manifestations related to hyperkalemia, including pacemaker non-capture, sensing failure and loss of delta wave in patients with Wolff-Parkinson-White syndrome [6]. There have been previous case reports of transient sinus conversion of permanent AF during treatment of hyperkalemia. The duration of maintenance of sinus rhythm after cardioversion varies among individuals. Two cases reported, describe the maintenance of sinus rhythm for 10 days [5] and 16 min [7] respectively. In the present case, sinus rhythm was maintained for 5 weeks after normalization of serum potassium. The exact mechanism for this conversion is not clearly known.

Correction of hyperkalemia results in shift of potassium inside cell and causes decrease in electrochemical gradient of sodium [8]. This results in inactivation of sodium channels responsible for start of the action potential and in turn affects the excitability of all myocardial cells. During treatment of hyperkalemia, intracellular influx of potassium prolongs the action potential duration and refractory period. The atrial cells are more susceptible to electrolyte imbalance than the sinus node in the refractory period because the phase 1 of the action potential in atrial cells is solely dependent of sodium influx [9]. Therefore hyperkalemia suppresses chaotic AF and causes transient sinus rhythm to recover during correction.

All ECG findings associated with hyperkalemia may be attributed to this mechanism. This mechanism bears many similarities to the pharmacological effects of amiodarone, which remains the most effective drug for maintaining sinus rhythm. The duration of maintenance after conversion to sinus rhythm remains undetermined and varies in different clinical scenarios. Therefore sinus rhythm obtained during treatment of hyperkalemia could eventually revert back to AF in hours to days or weeks.

### Conclusion

Apart from well known cardiac and electrocardiographic effects of hyperkalemia, iatrogenic or spontaneous correction of the same causes cardioversion of AF by change in the electrochemical milieu. It is thus mandatory to continue treatment of AF (anticoagulation) and maintain regular follow-up during this period following cardioversion.
